# Prolonged Seasonality of Respiratory Syncytial Virus Infection among Preterm Infants in a Subtropical Climate

**DOI:** 10.1371/journal.pone.0110166

**Published:** 2014-10-21

**Authors:** Chyong-Hsin Hsu, Chia-Ying Lin, Hsin Chi, Jui-Hsing Chang, Han-Yang Hung, Hsin-An Kao, Chun-Chih Peng, Wai-Tim Jim

**Affiliations:** 1 Department of Pediatrics, Division of Neonatology, Mackay Memorial Hospital, Taipei, Taiwan; 2 Department of Pediatrics, Division of Infectious Disease, Mackay Memorial Hospital, Taipei, Taiwan; Kliniken der Stadt Köln gGmbH, Germany

## Abstract

**Objective:**

There is limited epidemiological data on the seasonality of respiratory syncytial virus (RSV) infection in subtropical climates, such as in Taiwan. This study aimed to assess RSV seasonality among children ≤24 months of age in Taiwan. We also assessed factors (gestational age [GA], chronologic age [CA], and bronchopulmonary dysplasia [BPD]) associated with RSV-associated hospitalization in preterm infants to confirm the appropriateness of the novel Taiwanese RSV prophylactic policy.

**Study Design:**

From January 2000 to August 2010, 3572 children aged ≤24-months were admitted to Taipei Mackay Memorial Hospital due to RSV infection. The monthly RSV-associated hospitalization rate among children aged ≤24 months was retrospectively reviewed. Among these children, 378 were born preterm. The associations between GA, CA, and BPD and the incidence of RSV-associated hospitalization in the preterm infants were assessed.

**Results:**

In children aged ≤24 months, the monthly distribution of RSV-associated hospitalization rates revealed a prolonged RSV season with a duration of 10 months. Infants with GAs ≤32 weeks and those who had BPD had the highest rates of RSV hospitalization (*P*<0.001). Preterm infants were most vulnerable to RSV infection within CA 9 months.

**Conclusions:**

Given that Taiwan has a prolonged (10-month) RSV season, the American Academy of Pediatrics' recommendations for RSV prophylaxis are not directly applicable. The current Taiwanese guidelines for RSV prophylaxis, which specify palivizumab injection (a total six doses until CA 8–9 months) for preterm infants (those born before 28^6/7^ weeks GA or before 35^6/7^ weeks GA with BPD), are appropriate. This prophylaxis strategy may be applicable to other countries/regions with subtropical climates.

## Introduction

Respiratory syncytial virus (RSV) is the major pathogen of acute lower respiratory tract infection (ALRTI) in infancy and childhood [1], [2]. Of note, premature infants are ten-fold more likely than term infants to develop complicated RSV [3] and experience higher rates of hospitalization and mortality [4]. As there is no effective etiopathogenetic treatment once an infant is infected by RSV, effective RSV prophylaxis is extremely important [5].

Since 1998, the American Academy of Pediatrics (AAP) has recommended the use of palivizumab for passive immunization against RSV [6]. The AAP recommendations account for seasonality of RSV infection ie, in temperate climates, RSV infection rates typically peak during the cold season, whereas in tropical climates RSV infection rates typically peak during the rainy season [7]. To date, however, there is limited information regarding RSV seasonality in subtropical climates [6], [8]. As RSV surveillance is a globally important issue, a thorough understanding of RSV epidemiology in subtropical climates, such as that in Taiwan, is important for the optimization of global RSV prevention strategies. The current Taiwanese recommendations (published in 2010 December) for RSV prophylaxis specify six doses of palivizumab, targeting preterm infants born before 28^6/7^ weeks gestational age (GA) or those born before 35^6/7^ weeks GA with bronchopulmonary dysplasia (BPD), until a chronologic age (CA) of 8–9 months.

The purpose of this study was to determine the seasonality of RSV infection among children aged ≤24 months in Taiwan, a subtropical area. We also examined the effects of gestational age (GA), CA, and BPD on the incidence of RSV infection in preterm infants to confirm the appropriateness of the novel RSV prophylactic policy for premature infants in Taiwan.

## Methods

### Study Design and Data Collection

This retrospective single-center cohort study was conducted at Taipei Mackay Memorial Hospital, a tertiary medical center serving the greater Taipei metropolitan area in Northern Taiwan. Eligible participants were children aged ≤24 months who had a discharge diagnosis of RSV-associated bronchiolitis and/or pneumonia (ICD-9 CM Codes 466.11, 480.1, or 079.6) from January 2000 to August 2010. Preterm infants were included in the study if they were born in Taipei Mackay Memorial Hospital, had a GA <37 weeks, and were discharged alive from the neonatal intensive care unit (NICU) from 1 January 2000 to 31 August 2010. Prematurity was defined as birth before 37 weeks of GA (ie, GA ≤36 weeks and 6 days) in accordance with ICD-9 codes 765.10∼765.19 and 765.01–765.09. Infants were excluded from the study if they had congenital heart disease, other than patent ductus arteriosus or a septal defect that was hemodynamically insignificant, or any congenital anomaly. Repeat admission infants were also excluded because repeated admission may be related to other potentially confounding factors (aside from GA, CA, and BPD) eg, the level of neutralizing antibodies in the serum, etc.

A case manager from the Premature Baby Foundation of Taiwan assisted with the contact of preterm babies with very low birth weight (≤1500 g), almost all of whom had regular outpatient department follow-up visits after their discharge from Taipei Mackay Memorial Hospital. Note: for reasons of convenience, most preterm infants return to Taipei Mackay Memorial Hospital for any additional care requirements after discharge.

The diagnosis of RSV infection was confirmed by examination of nasopharyngeal specimens using either RSV antigen-specific direct immunofluorescence assay or virus culture. We defined a respiratory illness as being attributable to RSV if the patient had RSV infection necessitating hospitalization and was for positive the RSV-specific antigen or had a positive virus culture between 7 days before and 3 days after admission [8]. As the incubation period for RSV is typically 3 to 5 days, patients who had symptoms that appeared ≥5 days after admission were considered to have nosocomial RSV infection [7]–[9]. In addition to RSV testing, patients underwent throat virus cultures after admission (no comorbid viruses were detected).

We obtained the following information from neonatal chart records: GA, birth weight, gender, dates of nursery admission and discharge, and time on oxygen in the NICU. The presence of BPD was also recorded. BPD was defined as persistent oxygen dependency 28 days after birth [10], the need for oxygen at 36 weeks postmenstrual age [11], and the presence of a characteristic chest roentgenographic finding in accordance with ICD-9 code 770.7. De-identified patient data were collated by a single neonatologist. All links between the final data analyzed and the original data were removed. The study was approved by the local Study Review Board and Ethics Committee of Taipei Mackay Memorial Hospital. Written informed consent was given by next of kin of the participants.

### Outcome Measures

The monthly incidence of RSV-associated hospitalization for children aged ≤24 months was calculated for the study period to determine the seasonal activity of RSV infection at Taipei Mackay Memorial Hospital. We used the surveillance model for seasonality proposed by the Taiwan Centers for Disease Control (CDC) (Taiwan-CDC) [12], [13] as well as the seasonality model of the US CDC [14] to determine RSV seasonality in Taiwan. The RSV season was defined as a period of two or more consecutive months in which the RSV-associated hospitalization rate was above the baseline rate. The baseline RSV-associated hospitalization rate was calculated by firstly determining the monthly RSV-associated hospitalization rate for children aged ≤24 months old as follows: 
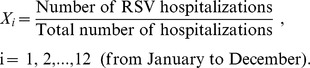



The average monthly RSV-associated hospitalization rate was then calculated as follows: 
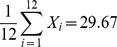
 (‰)

The months with RSV-associated hospitalization rates <29.67 ‰ were defined as “non-epidemic months”. The average monthly RSV-associated hospitalization rate of “non-epidemic months” was defined as the “baseline rate”.

Infants who were born preterm were categorized into three GA-based groups for comparison of RSV-related hospitalization: ≤28, 29–32, and 33–36 weeks.

Children who were born preterm with RSV infection were further stratified into three subgroups according to their CA of onset for comparison: ≤9, 10–15, and 16–24 months.

RSV-infected premature infants with underlying BPD were identified for subgroup data analyses.

### Statistical Analyses

Categorical variables are presented as counts and percentages, and were compared by chi-square test. Birth weight data are presented as median and full range. Statistical analyses were two-sided and carried out using SPSS software, version 20 (SPSS Inc., Chicago, IL). Statistical significance was indicated by *P*<0.05.

## Results

### Study Population

**Figure 1:**
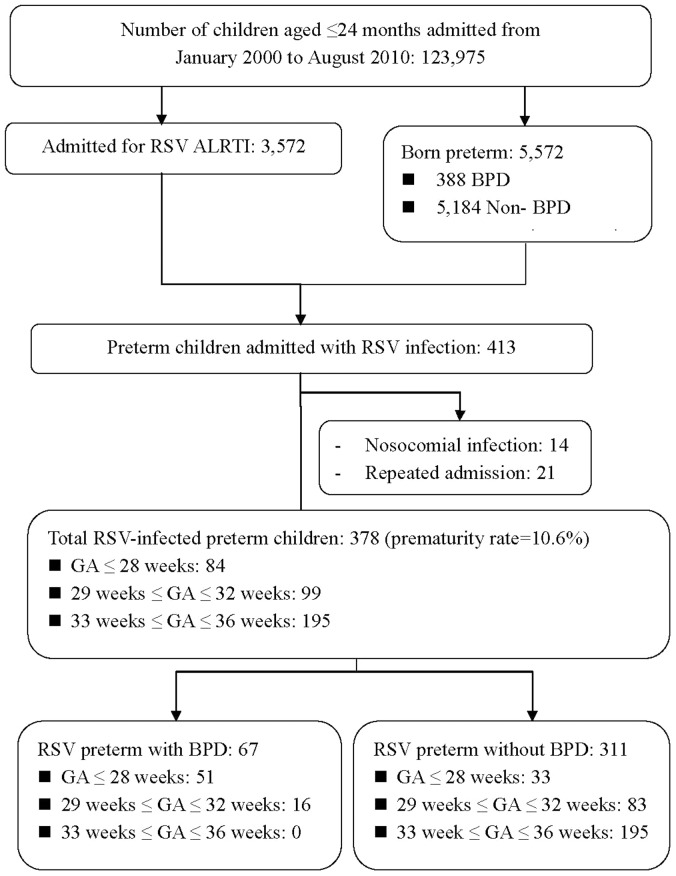
Flow chart of infants aged ≤24 months included in the study. ALRTI, acute lower respiratory tract infection; BPD, bronchopulmonary dysplasia; GA, gestational age; RSV, respiratory syncytial virus.

From January 2000 to August 2010, a total of 123,975 children aged ≤24 months were admitted to Taipei Mackay Memorial Hospital, 3,572 for RSV infection (Figure 1). Of these 5,572 children who were born premature (boys: n = 3048; girls: n = 2524; median birth weight: 1848 g [range: 522–3386 g]), 413 were admitted for RSV infection. After the exclusion of repeated admissions and infants who had nosocomial infections (Figure 1), 378 preterm infants were included in the study (boys: n = 230; girls: n = 148; median birth weight: 1859 g [range: 522–2864 g]). Of these infants, 67 had underlying BPD and 311 did not. Hence, 10.6% (378/3572) of the admissions due to RSV infection were preterm infants. The attributable mortality rate for RSV infection among the preterm infants was 7.9 ‰ (n = 3) in our study population.

### Monthly Incidence of RSV-Associated Hospitalization and Assessment of Seasonality

**Figure 2:**
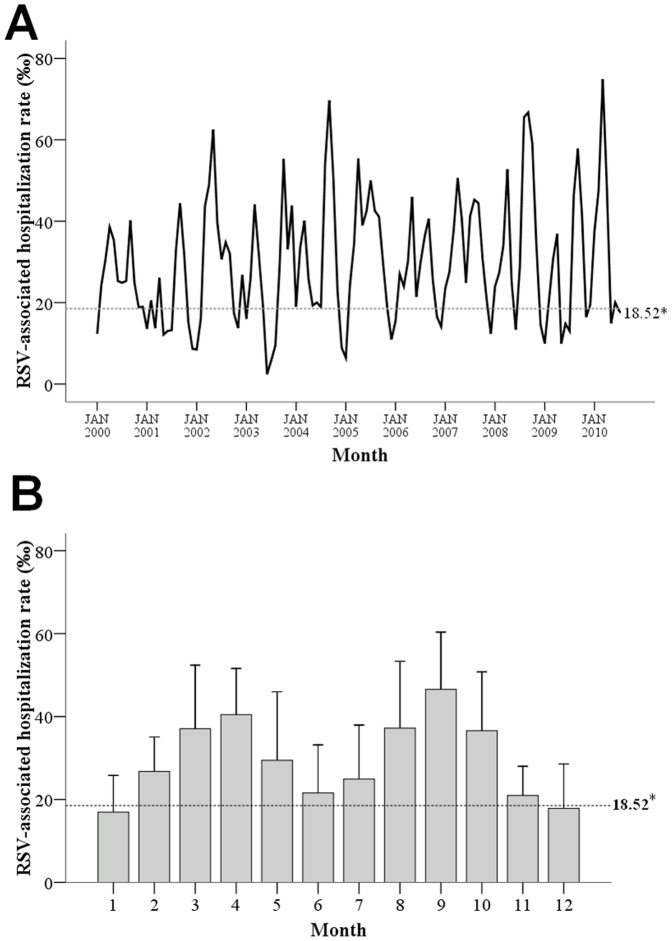
Monthly distribution of the RSV-associated hospitalization rate among all infants aged ≤24 months in the study (from January 2000 to August 2010). *The baseline rate of RSV infection was 18.52 ‰. RSV, respiratory syncytial virus. (A) Monthly RSV-associated hospitalization rates for the entire observation period. (B) Average monthly RSV-associated hospitalization rates from January to December for the observation period.

The monthly incidence of RSV-associated hospitalization among children aged ≤24 months in the study period is shown in Figure 2. Figure 2A summarizes the monthly RSV-associated hospitalization rate for the entire observation period. Figure 2B summarizes the average monthly RSV-associated hospitalization rate from January to December for the observation period. The baseline monthly RSV-related hospitalization rate was 18.52 ‰. The RSV season was defined as a period of two or more consecutive months, in which the RSV-associated hospitalization rate was above the basal rate. In our study, there was a prolonged, continuous RSV season lasting 10 months.

### The Impact of Gestational Age (GA) on RSV-Associated Hospitalization

The proportion of infants with RSV infection by GA (among the 5,572 infants who were born preterm) is summarized overall and by BPD status in Table 1. There were no differences in the rate of RSV infection by GA among infants who had underlying BPD. Of the non-BPD infants, those with GAs ≤28 weeks and 29–32 weeks had a significantly higher rate of RSV-associated hospitalization compared with those with a GA 33–36 weeks (*P*<0.001).

**Table 1:** Respiratory syncytial virus infection by gestational age for infants aged ≤24 months who were born premature (overall and by bronchopulmonary dysplasia status).

Infants	Gestational Age			*P* Value
	≤28 weeks	29–32 weeks	33–36 weeks	
Overall (378/5572), n/N (%)	84/584 (14.4)*	99/935 (10.6)*	195/4053 (4.8)	<0.001
With underlying BPD (67/388), n/N (%)	51/301 (16.9)	16/83 (19.3)	0/4 (0)	0.825
Without underlying BPD (311/5184), n/N (%)	33/283 (11.6)*	83/852 (9.7)*	195/4049 (4.8)	<0.001

BPD, bronchopulmonary dysplasia.

N  =  total premature babies (by gestational age); n  =  RSV-infected premature babies (by gestational age).

*Indicates a significant difference compared with gestational age 33–36 weeks.

### Effect of BPD on RSV Infection

Of the 5,572 premature infants, 388 had BPD and 5184 did not. A significantly higher proportion of infants who had BPD experienced RSV infection compared infants who did not have BPD (67/388; 17.3% vs 311/5184; 6.0%, *P*<0.001).

### Trends in RSV-Related Hospitalization by CA and Prematurity

**Figure 3:**
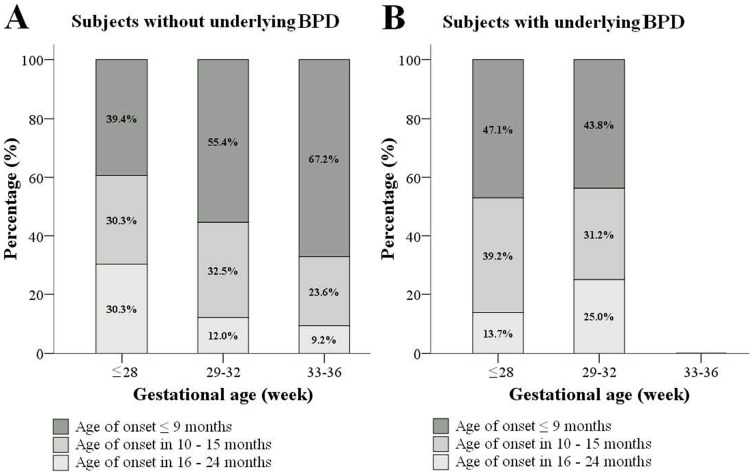
Percentage of infants (born premature) aged ≤24 months-with respiratory syncytial virus infection by age of onset (chronologic age), by gestational age, and BPD status (A: without BPD, n = 311; B: with BPD, n = 67). Note: there were no infants with underlying BPD at gestational age 33–36 months. BPD, bronchopulmonary dysplasia.

The proportion of infants hospitalized due to RSV infection by CA of onset (categorized by GA and BPD status) is presented in Figure 3. As most extremely low birth weight preterm infants (birth weight ≤1000 g) and very low birth weight infants (birth weight ≤1500 g) usually remained in hospital for 2–3 months after birth, we used a CA of 9 months (ie, almost 6 months after discharge) as the first cut-off point, and thereafter stratified the cut-off points by 6-month blocks to assess the timing of when preterm neonates were most vulnerable to RSV infection after discharge. Therefore, RSV-associated hospitalization rates were analyzed for infants grouped into three age cohorts: (≤9 months, 10 to 15 months, and 16 to 24 months). Moreover, the preterm infants with RSV with and without BPD were divided into 3 GA categories to facilitate GA comparisons: ≤28 weeks, 29–32 weeks, and 33–36 weeks.

#### Overall

Overall, 58.5% (221/378) of infants admitted due to RSV infections experienced onset within 9 months, 28.5% (108/378) experienced onset within 10–15 months, and 12.9% (49/378) experienced onset within 16–24 months. Regardless of BPD status and GA, RSV infection most frequently occurred within 9 months, followed by 10–15 months, and 16–24 months.

#### Without underlying BPD

The results for infants without underlying BPD are summarized in Figure 3A. Regardless of GA, RSV infection was most common within 9 months, followed by 10–15 months, and then 16–24 months.

#### With underlying BPD

The results for infants with underlying BPD are summarized in Figure 3B. There were no infants born at GA 33–36 weeks with BPD. Regardless of GA, RSV infection was most common within 9 months, followed by 10–15 months, and then 16–24 months.

## Discussion

RSV-prophylaxis strategies are globally important given that RSV circulates throughout the world. To the best of our knowledge, this is the first population-based, retrospective cohort study demonstrating prolonged seasonality of RSV infection (duration  = 10 months) among children aged ≤24 months in a subtropical climate. This is different from RSV epidemiology data obtained in the United States, Canada, and European countries. The prolonged RSV seasonality makes the establishment of an RSV prevention policy challenging because one of the most important elements of the current RSV prophylactic guideline recommended by AAP is the existence of a distinct RSV season for 5 months from November to March (or from December to April) in temperate climate zones [15]. Other countries located in subtropical climate regions, such as Hong Kong, Northern Vietnam, and southern China [16], [17] are also faced with the potential problem of RSV being present throughout the year, which complicates the development of effective prophylactic programs.

Taiwan is located at 23°0′N∼25°5′N latitude and 120° 39' E∼121° 33' E longitude; hence, the entire country is located in a subtropical climate region. Our result confirmed that there is a prolonged RSV season in Taiwan. In a previous report, Huang et al [6]reported that there was RSV infection year-round in Taiwan. Two previous retrospective studies [3], [18] also reported an apparent biannual pattern of RSV infection in Taiwan. The explanation for the disparate study findings may relate to differences in study design, unequal-aged based populations, population characteristics, and sample size. The prolonged seasonality in our study may be explained by the lack of marked seasonal changes in temperature and the lack of any profound effect of rainfall in Taiwan [19].

Given our finding of prolonged RSV seasonality, the AAP guideline on RSV prophylaxis with palivizumab are not directly applicable in Taiwan. The AAP guidelines specify a total of 5 doses (as the maximum) of monthly palivizumab to prevent RSV infection because the average duration of RSV season in temperate climates is 5 months [15]. The AAP guidelines also specify that palivizumab should be given during the RSV season to protect high-risk groups such as premature infants (GA ≤28^6/7^ weeks) or infants with BPD. Such an approach is not practical in a subtropical area with an RSV season lasting 10 months. Indeed, high-risk preterm infants would require 10 doses of palivizumab during the RSV season in Taiwan according to the AAP guidelines; this is not economically feasible. Hence, factors aside from seasonality must be considered in developing a reasonable and practical prophylaxis strategy in Taiwan (and presumably other countries in subtropical climate regions). Our findings suggest that GA, CA, and BPD are three important factors that should be considered in the establishment of cost–effective palivizumab prophylaxis strategies.

Our finding that preterm infants who have BPD have an increased risk for RSV infection are consistent with those determined in the IMpact RSV and other studies [5], [18], [20]–[22]. Our findings are also in keeping with those of the IRIS study group [21] who reported that preterm infants with either a GA ≤32 weeks or those with BPD have a very high risk of RSV-associated hospitalization. Of note, we found that CA ≤9 months was the most susceptible period for RSV infection among preterm infants in a subtropical climate with prolonged RSV seasonality. Only one previous report has examined this relationship and found that RSV-related hospitalization was most commonly observed among (predominantly term-born) infants aged 3–6 months [23]. Different to the findings from the IRIS and FLIP studies, which indicate that the risk of RSV infection is highest among infants with a CA <3 or 6 months at the start of the RSV season, our findings suggest that once preterm infants are discharged, monthly palivizumab should be administered for a total 6 doses until CA 8–9 months. There are several potential explanations for our finding regarding CA and the risk of RSV infection. First, preterm infants typically remain in the intensive care unit during the first 2–3 months after birth, so the risk of infection is highest within the first 6 months after discharge ie, 8–9 months of CA. Second, before CA 9 months, parents may become less vigilant about the potential risks of infection due to the relatively improved physiological conditions of their premature infants. Third, infants with underlying BPD (or other co-morbidities) often require frequent outpatient follow-up visits or interventions before CA 9 months, which may increase their likelihood of exposure to RSV. Finally, in Taiwan, infants' mothers return to work around this time and infants are typically cared for by babysitters thereafter. These babysitters usually look after more than one infant, thus increasing the risk of cross-infection. Our findings indicate that CA ≤9 months is a critical period for re-admission of preterm infants; hence, the first dose of palivizumab should be given 3–4 days before discharge and monthly thereafter until CA 8–9 months.

Although we employed a number of measures to minimize potential bias, our study does have several limitations. First, palivizumab was not widely available until 2010 December in Taiwan; hence, we could not conduct a prospective study to assess seasonality before 2010. Widespread prophylaxis with palivizumab from 2010 will make it difficult to collect the necessary epidemiologic data to analyze the risk factors for RSV-induced ALRTI in the future. For the same reason, there was no control group in our study. Despite being retrospective in nature, our study did encompass a significant (10-year) period of time and thus, we believe, contributes important epidemiological information. Second, although the RSV screening method used had very high sensitivity (93%) and specificity (98%), it is possible that some relevant cases of RSV infection were missed. Third, not all infants with ALRTI underwent RSV rapid test or throat virus culture. Hence, the RSV infection rate may have been underestimated. Fourth, as this study was carried out at a single institution, an additional population-based study acquiring data from multiple centers is warranted to confirm our findings and provide valuable information that could be used to optimize RSV prophylaxis. Despite the aforementioned limitations, we suggest that the data presented herein are extremely important, and provide a reasonable representation of RSV infection among children aged ≤24 months in a subtropical climate.

In our study, both GA≤28 weeks and GA 29–32 weeks were high-risk groups for RSV infection. Since December 2010, the Taiwan Society of Neonatology advised the Bureau of National Health Insurance in Taiwan to provide a total six monthly doses of palivizumab injections to premature infants with a GA ≤28^6/7^ weeks or prematurity with BPD and born before GA 35^6/7^ weeks, including the first dose before discharge and continuing until five months after discharge. Therefore, eligible infants will be protected by CA of 8–9 months, the most critical period of susceptibility to RSV infection. The current policy in Taiwan, subtropical climate country with prolonged RSV seasonality, is appropriate and may serve as a reference for other countries in subtropical climates in which the AAP guideline may not be applicable for RSV prophylaxis.
